# Hepatic sclerosed hemangioma and sclerosing cavernous hemangioma: a radiological study

**DOI:** 10.1007/s11604-021-01139-z

**Published:** 2021-05-27

**Authors:** Cuiyu Jia, Guangxue Liu, Xinxin Wang, Dawei Zhao, Ruili Li, Hongjun Li

**Affiliations:** 1 Department of Radiology, Beijing YouAn Hospital, Capital Medical University, Beijing, 100069 China; 2 Department of Natural Medicines, School of Pharmaceutical Sciences, Peking University Health Science Center, Beijing, 100191 China; 3 Department of Pathology, Beijing YouAn Hospital, Capital Medical University, Beijing, 100069 China

**Keywords:** Hepatic sclerosed hemangioma, Sclerosing cavernous hemangioma, Computed tomography, Magnetic resonance imaging, Diagnosis

## Abstract

**Purpose:**

To investigate and compare the CT and MRI features of hepatic sclerosed hemangioma (HSH) and sclerosing cavernous hemangioma (SCH).

**Materials and methods:**

Twelve HSH cases and 36 SCH cases were included, the imaging findings on CT (9 HSH and 34 SCH) and MRI (8 HSH and 10 SCH) were analyzed. Qualitative image analysis included the location, size, shape, capsular retraction, density, calcification, signal intensity on T_1_-weighted image (T_1_WI) and T_2_-weighted image (T_2_WI), presence of diffusion restriction, apparent diffusion coefficient (ADC) map, transient hepatic attenuation difference around the lesion, and the dynamic enhancement patterns.

**Results:**

The presence of liver cirrhosis in patients with HSH (3/12) was higher than SCH (1/36) (*P* = 0.043). The morphology appearance before enhancement showed no significant difference between HSH and SCH. Moreover, SCH had a stronger trend of centripetal enhancement patterns of cavernous hemangiomas (83.3%) compared to HSH (25%) (*P* < 0.001). Due to more frequent atypical enhancement features, containing rim-like enhancement, no enhancement, and peripheral heterogeneous enhancement, the misdiagnosis rate of HSH (75%) was significantly higher than that of SCH (16.7%) (*P* < 0.001). Furthermore, the ADC values of HSH and SCH were both higher than that of the surrounding liver parenchyma (*P* = 0.009, *P* = 0.002); however, there was no significant difference in ADC values between themselves (*P* = 0.613).

**Conclusion:**

SCH showed the same trend of centripetal enhancement characteristics as typical hemangioma, while HSH exhibited atypical enhancement features due to complete sclerosis. Higher ADC values might contribute to the identification of atypical HSH and SCH from malignancies.

## Background

Hepatic hemangiomas, as the most common benign liver tumor, could be categorized into small capillary hemangiomas, larger cavernous hemangiomas, hepatic sclerosed hemangioma (HSH) and sclerosing cavernous hemangioma (SCH) according to the number of fibrous tissues [1]. Typical hepatic hemangiomas are relatively easier to be diagnosed than atypical ones because of the unique enhancement characteristics [2, 3]. There are two key enhancement patterns that have long been considered as the hallmarks of hemangiomas on dynamic imaging [4]. One pattern demonstrates peripheral nodular enhancement in the arterial phase with centripetal filling in the portal venous phase, and persistent enhancement in the delayed phase [5, 6]. The other is the flash-filling pattern, which shows vivid homogeneous enhancement in the arterial phase, and absence of noticeable contrast washout in the late interstitial phase [7, 8].

HSH and SCH are extremely rare types of hepatic hemangiomas, mainly composed of hyalinization and fibrosis resulting from degenerative changes. SCH is defined by increasing fibrosis and hyalinization due to partially denatured changes in a cavernous hemangioma, while HSH is characterized by extensive fibrosis and almost completely obliterated vessels [9, 10], which represents the end stage of involution [3, 11]. HSH and SCH are sometimes difficult to be distinguished from hepatic malignancies, such as hepatocellular carcinoma (HCC) [12, 13], scirrhous hepatocellular carcinomas [14, 15], cholangiocarcinoma [15–18] and hepatic metastases [11, 19–22], for the lack of typical imaging appearance of cavernous hemangioma. Most of the tumors reported previously were resected due to preoperative misdiagnosis as hepatic malignancies. Therefore, this is an important aspect to be considered before planning invasive treatment is planned.

To date, only a few case reports of HSH and SCH have been recorded in the literature [11–22]. The case series of HSH have been documented only in a few studies, which were all limited by small sample sizes [23–25]. Moreover, no case series of SCH or study focusing on the differences of imaging features between HSH and SCH have been reported. In this study, the imaging manifestations of 12 cases of HSH and 36 cases of SCH confirmed by pathology were retrospectively analyzed to improve the understanding of the two diseases by comparing their imaging characteristics, so as to improve the diagnostic level and the differential diagnosis level of malignant tumors.

## Materials and methods

### Study population

**Fig. 1:**
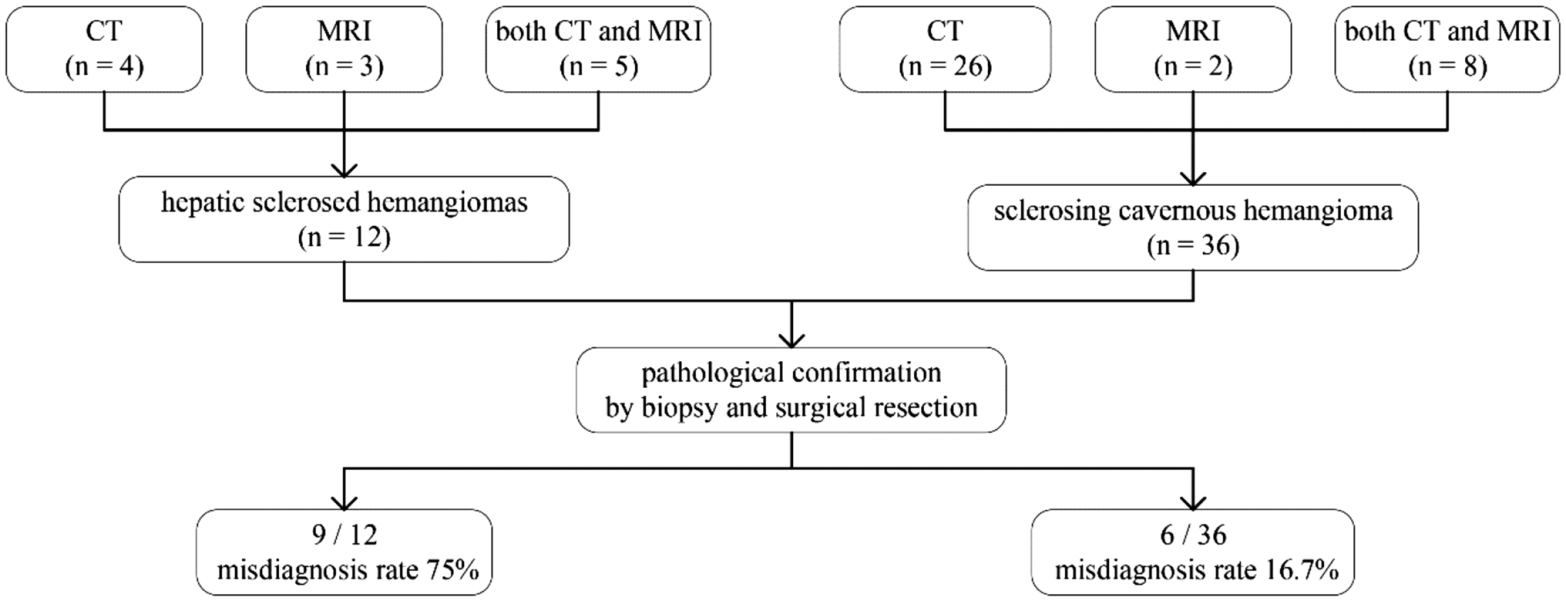
The flowchart of patients

This retrospective study was approved by the Ethics Committee of Beijing YouAn Hospital, Capital Medical University, and the informed consent was waived. Through a search of pathology reports from January 2010 to April 2019, a total of 12 consecutive cases of HSH and 36 cases of SCH were included and the flowchart of patients was shown in Fig. 1. All patients had available enhanced CT or MRI data within 30 days before surgery and biopsy. The general clinical characteristics of all patients including age, sex, hepatitis B or C, presence of liver cirrhosis, known malignant tumor, level of alpha-fetoprotein (AFP) and carbohydrate antigen 199 (CA199), imaging technique, methods of pathological confirmation, preoperative diagnosis and misdiagnosis rate were shown in Table 1.

**Table 1:** General clinical characteristics of study patients

	Hepatic sclerosed hemangioma (*n* = 12)		Sclerosing cavernous hemangioma (*n* = 36)	*P* value
Age (years)	50.5 (17–63)		48 (28–60)	0.401^†^
Sex (male)	66.7% (8/12)		30.6% (11/36)	0.041^‡,^*
Hepatitis B virus	33.3% (4/12)		16.7% (6/36)	0.241^‡^
Hepatitis C virus	0		0	
Liver cirrhosis	25% (3/12)		2.8% (1/36)	0.043^‡,^*
Known malignant tumor	Hepatocellular carcinoma (1) Cholangiocarcinoma (1)		Hepatocellular carcinoma (1)	0.150^‡^
Elevated of AFP	0		8.3% (3/36)	0.563^‡^
Elevated of CA199	25% (3/12)		5.6% (2/36)	0.092^‡^
Imaging technique	CT (4); MRI (3); CT + MRI (5)		CT (26); MRI (2); CT + MRI (8)	
Methods of pathological confirmation				0.018^‡,^*
Biopsy	50% (6/12)		13.9% (5/36)	
Surgical resection	50% (6/12)		86.1% (31/36)	
Misdiagnosis rate	75% (9/12)		16.7% (6/36)	0.000^‡,^*

*Significant level *P* < 0.05

^†^Independent sample Student *t* test

^‡^Fisher exact test

### CT protocol

CT imaging was performed using 64-detector row CT scanners (GE Lightspeed VCT, USA). The parameters were shown as follows: detector collimation, 0.625–1.25 mm; tube current, 380 mA; tube voltage, 120 kV; slice thickness, 5 mm; and pitch, 5 mm. Patients underwent a four-phase CT scan of the liver, including a non-contrast scan phase, an arterial phase, a portal venous phase, and a delayed phase. An iodine contrast agent (370 mg I/ml of Iopromide370, Schering, Berlin, Germany) was administered at a dose of 1.5 ml/kg at a rate of 3 ml/s via a mechanical power injector using a 20-gauge intravenous cannula placed in the antecubital vein. A smart prep contrast medium tracking technique was used during the arterial phase. When the CT value of the abdominal aorta reached or surpassed the threshold (150 HU), the scan was triggered. The venous phase was 65–70 s, and the delayed phase was 180–300 s. The thickness of the reconstructed image was 0.625 mm, and multiplanar reconstruction (coronal section) was performed.

### MR imaging protocol

MR imaging was performed with a 3.0-T MR scanner (TIM TRIO; Siemens, Erlangen, Germany) using a 32-channel body coil. The protocol consisted of T_1_-weighted (turbo-fast low angle shot, breath-hold scanning) (repetition time (TR)/echo time (TE) of 110.00/2.46 ms, slice thickness and gap of 5/1.5 mm, matrix size of 320 × 154, and field of view (FOV) of 440 × 640 mm), T_2_-weighted (single excitation half Fourier collection fast spin-echo sequence, breath-hold scanning) (TR/TE of 1200/88 ms, slice thickness and gap of 5/1.5 mm, matrix size of 384 × 200, and FOV of 616 × 768 mm), diffusion-weighted imaging (DWI) (*b*-values of 0, 150 and 800 s/mm^2^) with echo-planar imaging sequence, and Gadobenate Dimeglumine (Gd-BOPTA) enhanced scan. Gd-BOPTA was administered at a dosage of 0.2 mmol/kg at a rate of 2 ml per second. MR scans were obtained at the early arterial phase (22 s), late arterial phase (44 s), portal venous phase (60 s), and equilibrium phase (3–10 min) after administered the contrast agent. In this study, the hepatobiliary phase was set at a delay of 100–120 min [26], which was not a routine scan in our institution.

### Imaging analysis

Imaging findings on CT (9 HSH and 34 SCH), and MRI (8 HSH and 10 SCH) were analyzed. Qualitative image analysis included the location, size, shape, capsular retraction, density, calcification, signal intensity on T_1_-weighted image (T_1_WI) and T_2_-weighted image (T_2_WI), presence of diffusion restriction (defined as hyperintensity on DWI with a *b* value of 800 s/mm^2^, and iso-intensity to hypo-intensity on the apparent diffusion coefficient (ADC) map) [22], transient hepatic attenuation difference around the lesion, and the dynamic enhancement patterns (including typical enhancement pattern and atypical enhancement pattern). Typical enhancement pattern was defined as contrast agent centripetal filling in the arterial and venous phases, with the lesion completely or mostly filled in the delayed phases. Atypical patterns of enhancement included: (1) the trend of centripetal enhancement (it was explained as the enhancement around the lesion tended to expand inwards, but large non-enhancing areas of the central region in the delayed phase.); (2) peripheral linear and ring enhancement in the peripheral area in the arterial phase, until in the portal venous and delayed phase; (3) peripheral heterogeneous enhancement in the arterial phase, internal non-enhancement in the portal venous and delayed phase; (4) no enhancement.). The ADC values of the normal liver parenchyma and the hemangiomas were measured as quantitative analysis. The region of interest (ROI) (range 50–80 mm^2^) was drawn on the axial section of the largest cross-sectional area of the lesion to ensure uniformity of ROI placement. To minimize measurement errors, each radiologist performed two measurements on each lesion and then averaged them. A total of two radiologists with 10 and 23 years of abdominal imaging experience, respectively, read the images together. If there were any discrepancies, a consensus diagnosis was reached by consulting the superior doctors. All the reading radiologists were blinded to the final pathological diagnosis.

### Pathological examination

The specimens were subjected to histopathological examination with hematoxylin and eosin staining, followed by immunohistochemical examination. All pathological specimens were reviewed by an experienced pathologist with 22 years of expertise in hepatobiliary pathology. Pathological diagnostic criteria: SCH were explained as a localized degenerative change within a hemangioma due to increasing degree of fibrosis and thrombosis, infarction, or hemorrhage. HSH were used to describe complete degeneration, and the histopathological features are extensive fibrosis, hyaloid degeneration, increased elastic fibers, dystrophic calcifications, numerous thick-walled blood vessels, and significant stenosis or occlusion of the vascular lumen [9, 10].

### Statistical analysis

Demographic, clinical, and imaging data were assessed with IBM SPSS 20.0 (IBM Inc. Armonk, NY, USA). The Kolmogorov–Smirnov test was used to evaluate the normal distribution of continuous variables. Age and ADC values of the normal liver parenchyma and the hemangiomas were normally distributed, means ± standard deviations were present, and independent samples *t* test was used to analyze the differences between the two groups. Mann–Whitney *U* test was performed to compare the difference of tumor sizes between the groups and the medians (minimum, maximum) were present. For categorical variables, Fisher’s exact test was employed. *P* values < 0.05 were considered statistically significant.

## Results

### Clinical characteristics

The general clinical characteristics of patients are shown in Table 1. Both HSH and SCH were common in middle-aged people with median ages of 50.5 and 48, respectively. There were no significant differences between both groups in terms of ages, levels of AFP and CA199, and the incidence of hepatitis B/C or known malignant tumor. The presence of liver cirrhosis in patients with HSH (3/12) was higher than SCH (1/36) (*P* = 0.043), and HSH were predominantly male (66.7%), higher than SCH (30.6%) (*P* = 0.041). Furthermore, patients with HSH underwent biopsy (50%) more often than patients with SCH (13.9%) (*P* = 0.018). It was speculated that most HSH were considered to be malignant lesions based on the imaging findings, requiring further histology to determine the nature of the lesion. In contrast, most cases of SCH were surgically resected due to giant lesions, abdominal symptoms, or complications. The misdiagnosis rate of HSH (75%) was significantly higher than that of SCH (16.7%) owing to atypical imaging findings (*P* < 0.001), based on the evaluation of radiological reports. The characteristics of misdiagnosed cases are shown in Table 2.

**Table 2:** The clinical characteristics of misdiagnosed cases

Gender and age	Single or multiple	Known malignant tumor	HBV or HCV	Liver cirrhosis	Abnormal tumor markers	Imaging technique and enhancement patterns	Radiology diagnosis	Pathological diagnosis
F, 51	Single	N	N	N	N	MRI, rim-like enhancement	Probably malignancy	HSH
M, 17	Multiple	N	N	N	N	MRI + CT, no enhancement	Probably malignancy	HSH
F, 56	Single	N	N	N	N	CT + MRI, rim-like enhancement	Probably malignancy	HSH
F, 27	Multiple	N	N	N	N	MRI, no enhancement	Probably malignancy	HSH
M, 63	Single	N	N	N	CA199	CT + MRI, peripheral heterogeneous enhancement	ICC	HSH
M, 50	Multiple	N	HBV	Liver cirrhosis	CA199	CT + MRI, no enhancement	Probably malignancy	HSH
M, 53	Single	N	HBV	Liver cirrhosis	N	CT, no enhancement	Probably malignancy	HSH
F, 54	Single	ICC	HBV	N	CA199	CT, no enhancement	Metastasis	HSH
M, 52	Single	HCC	HBV	N	N	CT + MRI, rim-like enhancement	Metastasis	HSH
M, 51	Single	N	N	N	N	CT, peripheral heterogeneous enhancement	ICC	SCH
M, 48	Single	N	HBV	N	AFP CA199	CT + MRI, no enhancement	HCC	SCH
F, 34	Single	N	HBV	N	N	MRI + CT, wash-in and wash-out	HCC	SCH
F, 46	Single	N	N	N	AFP CA199	CT, no enhancement	Probably malignancy	SCH
M, 36	Multiple	N	N	N	N	CT + MRI, no enhancement	Probably malignancy	SCH
F, 60	Single	N	N	N	N	CT, no enhancement	Probably malignancy	SCH

*F* female, *M* male, *N* not found, elevated level of alpha-fetoprotein (AFP) and carbohydrate antigen 199 (CA199), *HSH* hepatic sclerosed hemangioma, *SCH* sclerosing cavernous hemangioma, *MRI + CT* MRI examination was first performed, then CT was performed for further differential diagnosis, *CT + MRI* CT first, then MRI for further differential diagnosis

### Location and morphologic appearance

**Fig. 2:**
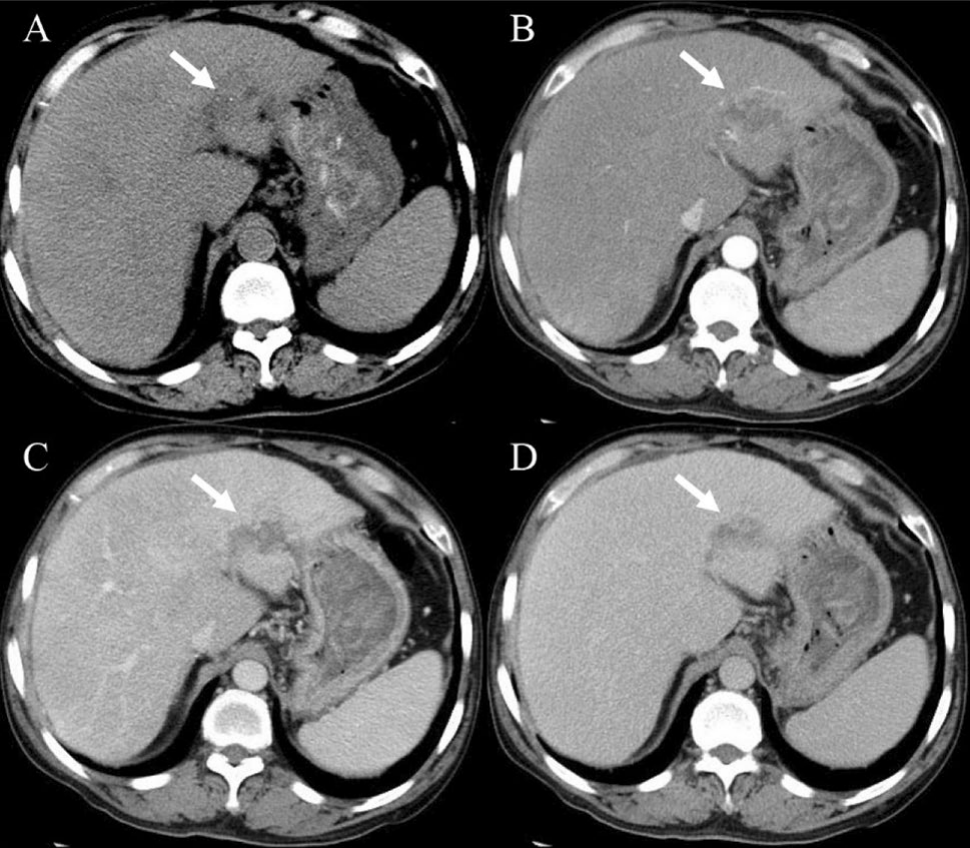
A case of hepatic sclerosed hemangioma mimicking intrahepatic cholangiocarcinoma (man, 63-years-old). Plain CT revealed a slightly low-density mass with dot-like calcification and irregular margin in segment 2 of the liver (**A**). The mass showed peripheral heterogeneous enhancement in the arterial phase (**B**), internal non-enhancement in the portal venous phase (**C**) and delayed phase (**D**) (arrows)

**Fig. 3:**
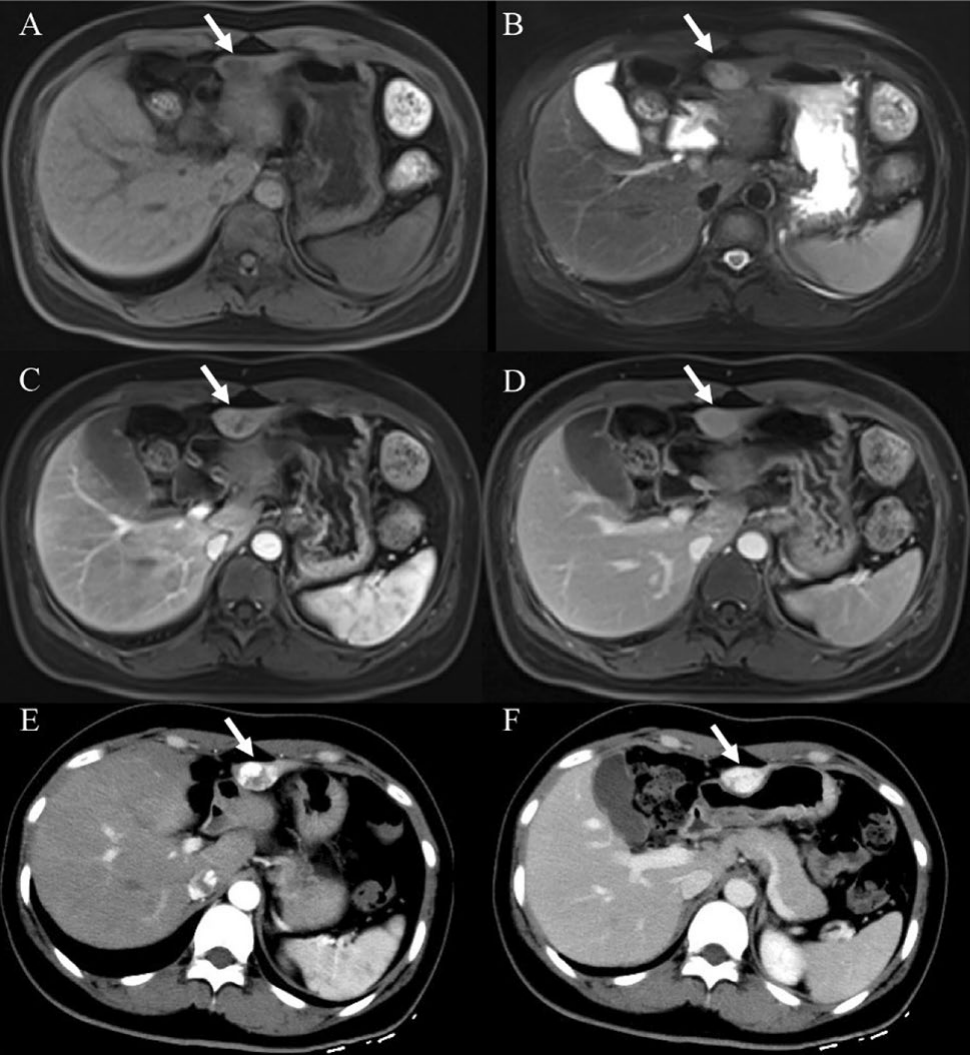
A case of sclerosing cavernous hemangioma mimicking hepatocellular carcinoma on MRI images (a 34-years-old female patient had chronic hepatitis B for more than 10 years). The mass was located in segment 3. It showed slightly hypointense on T_1_-weighted fat-suppressed image (**A**), slightly hyperintense on T_2_-weighted fat-suppressed image (**B**), heterogeneous enhancement in the arterial phase (**C**), and a subtle low-signal intensity with capsular enhancement in the delayed phase (**D**). Based on MRI findings, well-differentiated hepatocellular carcinoma was suspected. CT was further performed to rule out hemangioma. Enhanced CT demonstrated a typical nodular enhancement in the arterial phase (**E**) and continued filling in during the portal venous phase (**F**) (arrows)

**Fig. 4:**
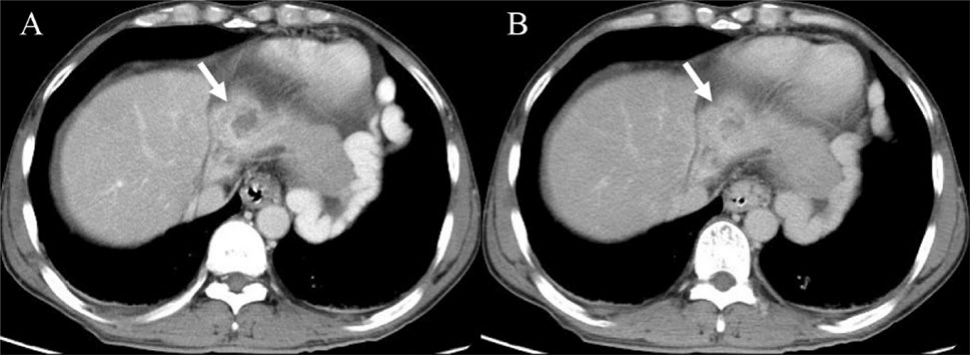
A case of sclerosing cavernous hemangioma mimicking intrahepatic cholangiocarcinoma (male, 51-years-old). Axial CT in the portal venous phase showed an irregular peripheral heterogeneous enhancement mass with capsular retraction, left lobe atrophy and mild biliary ductal dilatation in the left lobe (**A**), internal non-enhancement in the delayed phase (**B**) (arrows)

Before enhancement, the location and morphologic appearance showed no significant difference between the two groups (Table 3). Both HSH and SCH were more common in the right lobe of the liver. The median sizes of HSH and SCH were 3.9 cm and 7.5 cm, respectively. The irregular shape and heterogeneous density were the characteristic of most lesions, which was demonstrated with slightly low density on plain CT (Fig. 2A), slightly hypo-intensity on T_1_WI (Fig. 3A), and slightly hyperintensity on conventional T_2_WI (Fig. 3B). The capsular retraction (Fig. 4A) and calcification (Fig. 2A) were exhibited in a few lesions.

**Table 3:** Imaging features of sclerosed hemangioma and sclerosing hemangiomas

	Hepatic sclerosed hemangioma (*n* = 12)	Sclerosing cavernous hemangioma (*n* = 36)	*P* value
Location			0.510^‡^
Left lobe	33.3% (4/12)	47.2% (17/36)	
Right lobe	66.7% (8/12)	52.8% (19/36)	
Tumor size (cm)	3.9 (1–36.5)	7.5 (1–39)	0.323^†^
Tumor shape			0.316^‡^
Round	25% (3/12)	44.4% (16/36)	
Irregular	75% (9/12)	55.6% (20/36)	
Capsular retraction	8.3% (1/12)	5.6% (2/36)	1.000^‡^
Slightly low density on plain CT	88.9% (8/9)	79.4% (27/34)	1.000^‡^
Calcification	33.3% (3/9)	8.8% (3/34)	0.095^‡^
Heterogeneous density	55.6% (5/9)	58.8% (20/34)	1.000^‡^
Slightly hypointense on T_1_-weighted image	75% (6/8)	80% (8/10)	1.000^‡^
T_2_-weighted image			0.444^‡^
Hyperintense (similar to cerebro-spinal fluid)	12.5% (1/8)	0	
Slightly hyperintense	87.5% (7/8)	100% (10/10)	
Restriction on DWI	0	0	
Transient hepatic attenuation difference around the lesion	0	16.7% (6/36)	0.315^‡^
Dynamic enhancement patterns			0.000^‡,^*
Trend of centripetal enhancement accompanied by central non-enhancing areas	25% (3/12)	83.3% (30/36)	
Atypical	75% (9/12)	16.7% (6/36)	

*Significant level *P* < 0.05

^†^Mann–Whitney *U* test

^‡^Fisher exact test

### Enhancement characteristics

**Fig. 5:**
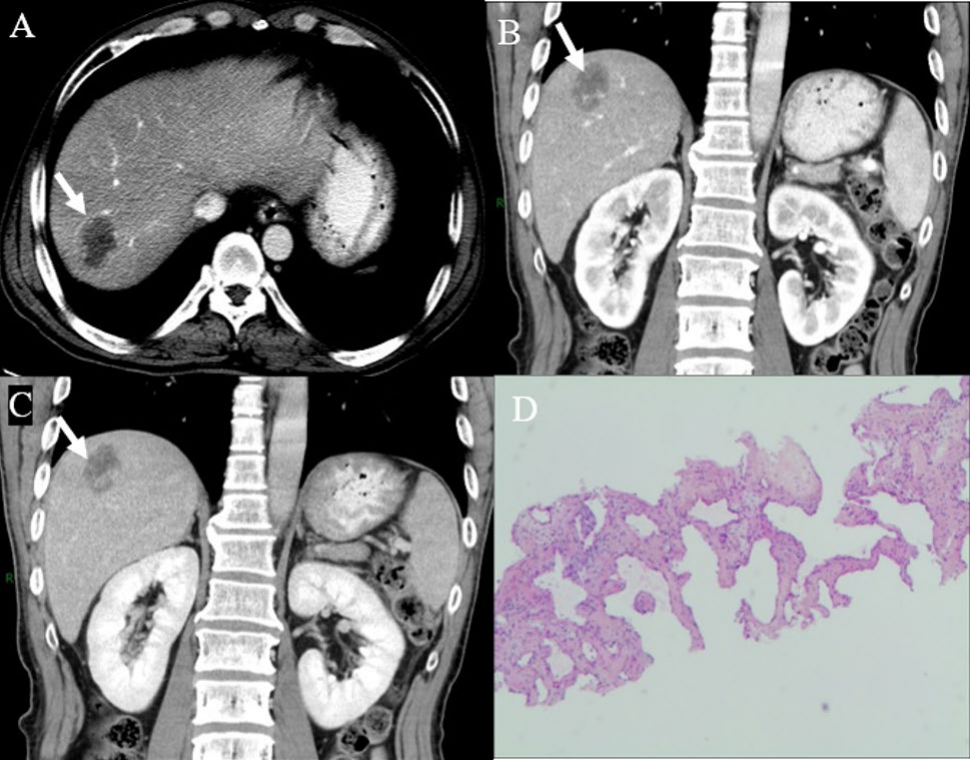
A case of sclerosing cavernous hemangioma was misdiagnosed as a malignant disease (A 48-years-old male patient, who had chronic hepatitis B for more than 20 years, referred to our hospital because of AFP elevation (183.8 ng/ml)). The lesion arose from segment 7 of the liver. Axial CT showed peripheral small nodular or dot-like enhancement in the arterial phase (**A**) (arrows). Coronal CT showed the trend of centripetal enhancement, the majority central non-enhancing areas in the portal venous phase and delayed phase (**B**, **C**) (arrows). Hematoxylin and eosin (H&E) staining (40 ×) (K) showed a large number of fibrous tissues, hyaline degeneration, and focal infarction

**Fig. 6:**
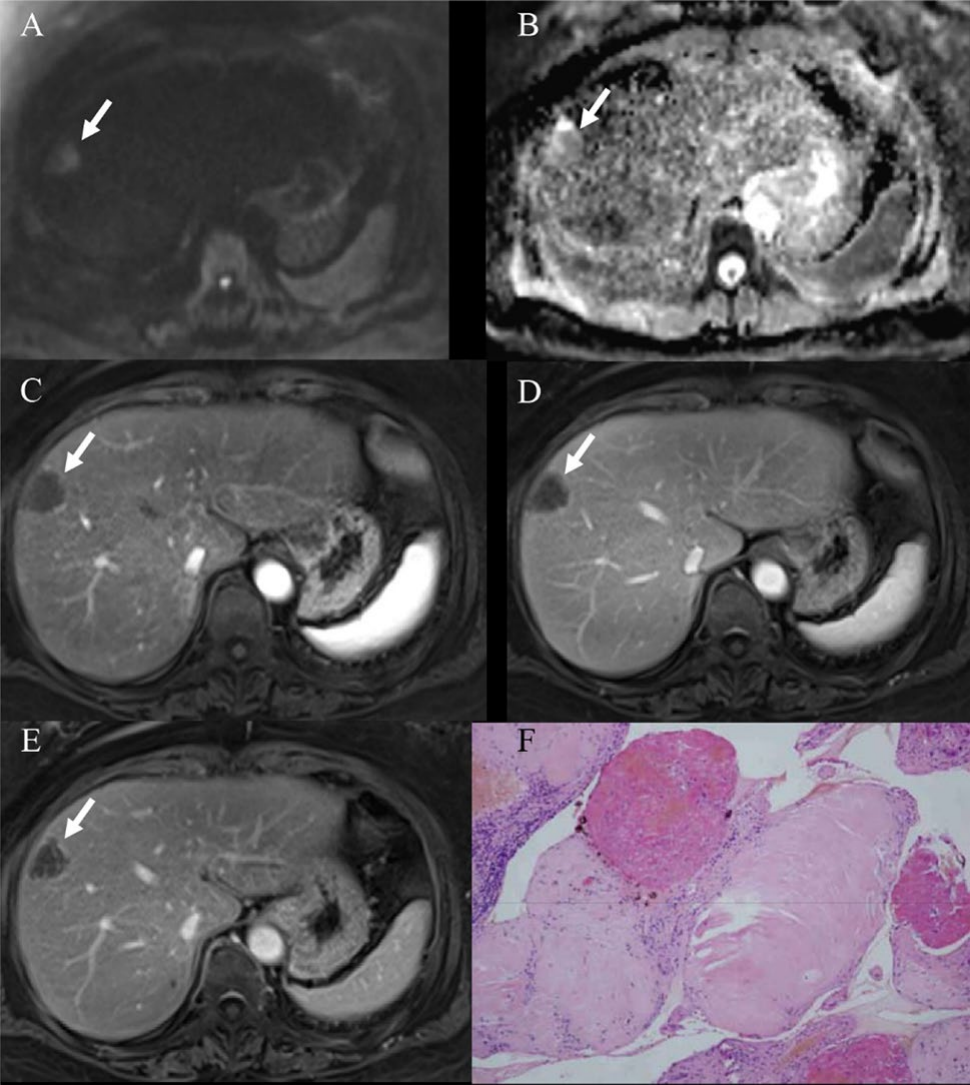
A case of the hepatic sclerosed hemangioma was misdiagnosed as a possible malignant disease (female, 56-years-old). The lesion arose from segment 8 of the liver. It showed slight hyperintense on diffusion-weighted image and isointense on ADC map, but the ADC value of the lesion was higher than the surrounding liver parenchyma, suggesting no diffusion restriction (**A**, **B**). Arterial phase (**C**), portal venous (**D**), and delayed phase (**E**) of enhanced MR images demonstrated ring enhancement (arrows). Fibrous connective tissue, sclerotic stroma, and hyaline degeneration areas were seen in the lesion in Hematoxylin and eosin (H&E) staining (100 ×) (**F**)

**Fig. 7:**
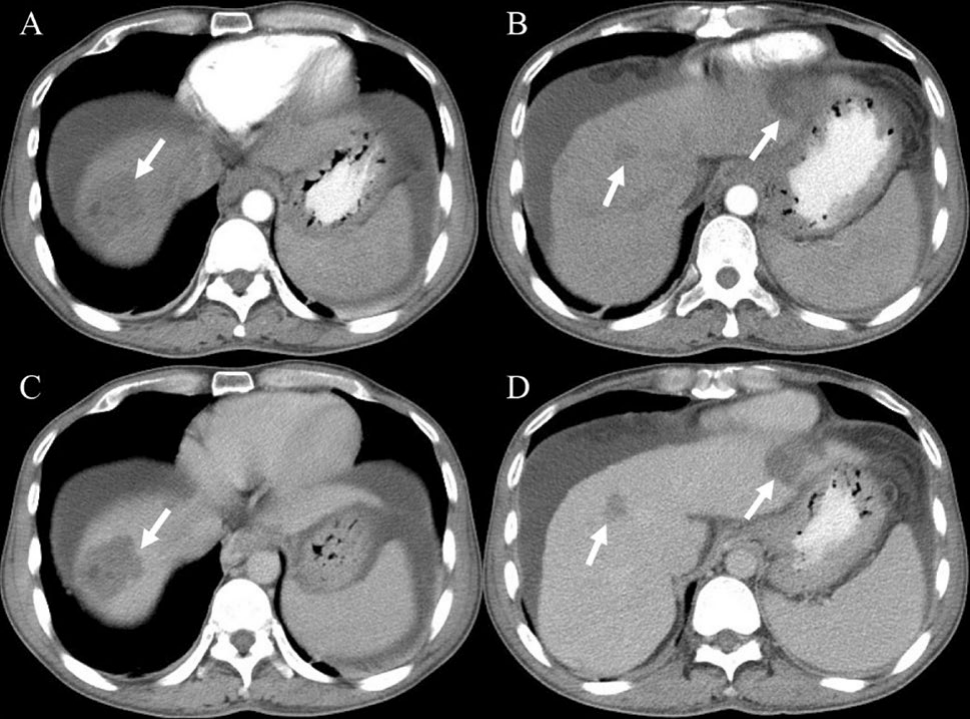
A case of multiple hepatic sclerosed hemangiomas was misdiagnosed as a possible malignant disease (male, 50-years-old). Axial CT in the arterial phase and delayed phase demonstrated multiple irregular heterogeneous hypodense lesions in the liver with no obvious enhancement (**A**–**D**) (arrows)

A higher tendency of trend of centripetal enhancement patterns were observed in SCH cases (83.3%) compared to HSH (25%) (*P* < 0.001; Table 3), which was illustrated with peripheral small nodular or dot-like enhancement in the arterial phase (Fig. 5A), slightly centripetal enhancement around the nodule in the portal venous phase (Fig. 5B), central non-enhancing areas in the delayed phase (Fig. 5C). Most HSH and a fewer of SCH cases exhibited atypical enhancement features, containing rim-like enhancement (Fig. 6C–E) in the delayed phase, no enhancement (Fig. 7A–D), and peripheral heterogeneous enhancement (Figs. 2B–D, 3C, D, 4A, B). A total of 16.7% SCH showed transient hepatic attenuation difference around the lesion in the arterial phase, and which was not found in all HSH cases (Table 3).

### Diffusion restriction and ADC values

The diffusion restriction on DWI (*b* = 800 s/mm^2^) was not found in all lesions (Fig. 6A, B). Furthermore, the ADC values of HSH and SCH were both higher than the surrounding liver parenchyma (*P* = 0.009, *P* = 0.002); however, there was no significant difference in the ADC values between HSH and SCH (*P* = 0.613) (Table 4).

**Table 4:** The ADC values of the hepatic sclerosed hemangioma, sclerosing cavernous hemangioma, and the adjacent liver parenchyma

	Hepatic sclerosed hemangioma	Sclerosing cavernous hemangioma	*P* value
ADC values of the lesions	(1.684 ± 0.476) × 10^–3^ mm^2^/s	(1.786 ± 0.370) × 10^–3^ mm^2^/s	0.613^†^
ADC values of the normal liver parenchyma	(1.159 ± 0.128) × 10^–3^ mm^2^/s	(1.305 ± 0.109) × 10^–3^ mm^2^/s	
*P* value	0.009^†,^*	0.002^†,^*	

*Significant level *P* < 0.05

^†^Independent samples Student t test

## Discussion

The incidence of hepatic hemangiomas in the general population is 1–20%, hepatic hemangiomas usually have typical imaging features, and it is not difficult to be diagnosed [20]. However, HSH and SCH are rare types of hepatic hemangiomas, and their imaging data are also limited. Previous isolated case reports found that their imaging appearances were similar to hepatic malignancies [11–22], which could result in misdiagnosis and unnecessary surgical resection. In this study, the imaging features and diagnostic experience of HSH and SCH were summarized retrospectively. The results indicated that most SCH exhibits the trend of centripetal enhancement characteristics and were correctly diagnosed. In contrast, HSH’s the enhancement patterns were variable and atypical, and a differential diagnosis of HSH from other malignant liver tumors may be proposed.

The clinical features and morphology appearance of HSH and SCH on plain CT were non-specific and insignificant for the identification of benign and malignant lesions. Both HSH and SCH were observed relatively predominantly in middle-aged people and occurring more frequently in the right lobe [10]. SCH was more frequent in women, while HSH was more common in men. Most of HSH and SCH exhibited irregular shapes and heterogeneous densities or signals, and demonstrated slightly low densities on CT, slightly hypo-intensity on T_1_WI, and slightly hyperintensity on conventional T_2_WI rather than homogenous hyperintensity similar to those of cerebrospinal fluid, caused by the long T_2_ relaxation time of its stagnant flowing oxygenated blood-filled vascular channels [27]. Obviously narrowed or obstructed vascular lumens, collagen depositions, and fibrous tissues could result in lower signals on T2WI compared to typical cavernous hemangiomas.

It should be noted that, although the enhancement characteristics of SCH were different from those of typical hemangiomas, most SCH exhibited the trend of centripetal enhancement characteristics, which could be illustrated by the punctate or small nodular edge enhancement during the arterial phase, centering, or spreading around the nodule during the venous phase and lasting until the delayed phase, as well as extensive internal unenhanced areas. These lesions could be characterized by unenhanced areas due to central or eccentric fibrous tissues, hyaline degeneration, and focal infarcts in the lesions. Understanding this enhancement pattern could improve the accuracy of diagnosing SCH, and observation was a prudent option of management.

The majority of HSH exhibited atypical enhancement characteristics, with little enhancement, or no enhancement during the arterial phase, only subtle linear marginal enhancement during the delayed phase, or peripheral heterogeneous enhancement with most lesions no enhancement. Previous studies also indicated that HSH lacked enhancement on enhanced CT and MR scans or during the arterial phase, and only exhibited linear enhancement in the peripheral area during the delayed phase or irregular peripheral enhancement [15, 23, 24]. The atypical enhancement patterns of HSH could be associated with the obliteration of vascular channels and extensive tissue fibrosis on pathology, developed from the center to the periphery of the hemangiomas and eventually involving the whole lesions [24, 28].

In general, HSH exhibited more atypical enhancement characteristics than SCH, which could be related to the degree of degeneration. HSH, used to describe complete degeneration, its histopathological features were extensive fibrosis, hyaloid degeneration, increased elastic fibers, dystrophic calcifications, numerous thick-walled blood vessels, and significant stenosis or occlusion of the vascular lumens [10]. SCH, however, was speculated to be a transitional stage of hemangioma degeneration, compared to HSH which was used to describe the developed stage of significant fibrosis and complete occlusion of the vascular lumens. Although the relationship between HSH and SCH was inconclusive, Makhlouf et al. stated that HSH was resulted from capillary hemangiomas, and SCH was developed from cavernous hemangiomas [10]. However, Shimada et al. reported one case of HSH developed from a cavernous hemangioma over 10 years of evolution. Further research was needed to clarify the origins and relationship between HSH and SCH [11].

In this study, some atypical hemangiomas were misdiagnosed as HCC, cholangiocarcinoma, metastatic tumors, and probably malignancies, especially when the patients had multiple lesions, or were accompanied by other medical histories, such as hepatitis B, cirrhosis, elevated AFP, or other malignant tumors. How to differentiate these atypical HSH and SCH from other hepatic malignancies was very important for clinical practices.

DWI has been widely used in identifying benign and malignant intrahepatic lesions [29, 30], but rarely applied to the identification of HSH and SCH. In this study, the DWI showed iso- or slightly high-signal intensity under the condition of high *b* value. Although some lesions on the ADC map showed iso-signal intensity, the ADC values of all the lesions were higher than the surrounding liver parenchyma, which also suggested that they may be benign lesions. Many hyalinized ingredients with the liquiform degeneration of HSH and SCH may be one of the reasons for high ADC values [31]. Similarly, some earlier studies have also reported that the ADC values of HSH and SCH were higher than those of the background livers [23, 31–33]. It could be speculated that the absence of diffusion restriction and the quantitative ADC values could be the important diagnostic clues to differentiate HSH and SCH from other hepatic malignant tumors based on these results.

There were several limitations to this study. First, the limited sample size of this study might have an adverse effect on the conclusion. Second, a possible limitation would be the fact that definite diagnosis was achieved only with biopsy in some cases and with resection in the others, although both methods were probably equally effective for diagnosis. Third, the ADC values varied greatly among individuals, and it was impossible to establish a unified standard to identify HSH and SCH based on ADC values in this study. However, a higher ADC value of the lesion could indicate a benign case by comparing the ADC value of its own liver. Fourth, the MR imaging features of HSH and SCH during the hepatobiliary phase could not be summarized for the lack of MR cases, and thus more cases should be evaluated in the future. Finally, this study focused on a comparative assessment of the imaging features of HSH and SCH, while the differences between HSH and malignant tumors were rarely mentioned, which might be more important for clinical practices and necessary to be clarified in the future.

## Conclusions

Knowledge of the imaging features of HSH and SCH can improve diagnostic confidence. Most SCH exhibits the trend of centripetal enhancement characteristics, though there are large non-enhancing areas in the center of the lesion, which are easy to differentiate from HSH and malignant tumors. A large proportion of HSH exhibit atypical enhancement features due to the complete of sclerosis, including rim-like enhancement, no enhancement, and peripheral heterogeneous enhancement. Higher ADC value is valuable for the differential diagnosis of HSH and SCH from malignancies.

## Data Availability

The datasets used and analyzed in this study are available from the corresponding author on reasonable request.

## Abbreviations

HSH: Hepatic sclerosed hemangioma
SCH: Sclerosing cavernous hemangioma
CT: Computed tomography
MRI: Magnetic resonance imaging
ADC: Apparent diffusion coefficient
HCC: Hepatocellular carcinoma
AFP: Alpha-fetoprotein
CA199: Carbohydrate antigen 199
DWI: Diffusion-weighted imaging
Gd-BOPTA: Gadobenate dimeglumine
T1WI: T1-weighted image
T2WI: T2-weighted image
